# Creation of Genetically Modified Adipocytes for Tissue Engineering: Creatine Kinase B Overexpression Leads to Stimulated Glucose Uptake and Mitochondrial Potential Growth, but Lowered Lipid Synthesis

**DOI:** 10.3390/life15050753

**Published:** 2025-05-08

**Authors:** Svetlana Michurina, Irina Beloglazova, Margarita Agareva, Natalia Alekseeva, Yelena Parfyonova, Iurii Stafeev

**Affiliations:** 1 Chazov National Medical Research Centre for Cardiology, Moscow 121552, Russia; michurinas192@gmail.com (S.M.); amarrgo1999@gmail.com (M.A.); yeparfyon@mail.ru (Y.P.); 2 Faculty of Fundamental Medicine, Lomonosov Moscow State University, Moscow 119991, Russia; 3 Faculty of Biology, Lomonosov Moscow State University, Moscow 119991, Russia; alekseevanv1011@mail.ru

**Keywords:** adipocytes, thermogenesis, creatine kinase B, glucose metabolism, lipid metabolism

## Abstract

Background: The global burden of obesity and type 2 diabetes mellitus is a significant contributor to mortality and disability in the modern world. In this regard, the modification of adipocyte metabolism has been identified as a promising approach to develop new genetic and cellular engineering therapeutics. In this study, we activate the expression of creatine kinase B (CKB), a key enzyme of a non-canonical futile cycle and the regulator of energy storage, to promote catabolic processes in mature adipocytes. Methods: The protein-coding sequence of CKB was amplified by PCR from *Mus musculus* brain mRNA. Lentiviral transduction was used to transfer the CKB sequence into mature adipocytes. Adipocyte metabolism was analyzed by radioisotope monitoring of labeled [3H]-2-deoxyglucose and [14C]-glucose. Confocal microscopy was applied to estimate lipid droplets morphology (BODIPY493/503 dye), mitochondrial membrane potential (JC-1 dye), and thermogenesis (ERthermAC dye). Results: After lentiviral delivery of the CKB-coding sequence, CKB mRNA level increased 75-fold and protein expression fivefold. CKB overexpression does not cause significant changes in lipid droplet morphology. Despite this, enhanced glucose uptake and reduced lipid synthesis under adrenergic stimulation are detected during CKB overexpression. CKB causes an increase in mitochondrial potential with no effect on thermogenesis in adipocytes. Conclusions: In this study, we have shown that CKB overexpression in mature adipocytes allows us to obtain adipocytes with high glucose uptake, potency of ATP synthesis, and suppressed lipogenesis. These genetically modified cells may potentially exhibit a favorable metabolic effect in the context of excessive nutrient utilization.

## 1. Introduction

Today, the consumption of ultra-processed foods and hypodynamia are leading the human population towards an obesity pandemic [1]. Obesity develops when there is a chronic imbalance between nutrient intake and energy expenditure. The first tissue to respond to excessive caloric consumption is adipose tissue. At the initial stage of metabolic overload, adipose tissue adapts by activating adipogenesis [2]. However, chronic caloric excess diminishes the adaptive capacity of adipose precursors, resulting in suppression of adipogenesis, adipocyte hypertrophy, and inflammation [3,4]. Altogether, these factors lead to the development of insulin resistance, a key molecular manifestation of type 2 diabetes. Thus, the control of adipocyte size and metabolism may be critical for the treatment of obesity and the prevention of type 2 diabetes.

According to the prevailing knowledge about energy balance, two therapeutic approaches are available for obesity treatment. The first approach involves the restriction of energy intake, exemplified by iSGLT2 drugs, which promote glucose excretion without tissue uptake [5]. The second approach entails the stimulation of energy expenditure. In adipocytes, this can be achieved through thermogenesis, a process that uncouples respiration and ATP synthesis in mitochondria, resulting in the release of heat due to enhanced mitochondrial electron transport chain activity. This approach has been a subject of discussion in the literature since 2014 and has undergone significant development in the last decade [6,7,8]. Thermogenesis induction can be achieved through two general strategies: pharmacological and genetic approaches. The spectrum of pharmacological agents that serve as thermogenic inducers is extensive. These include hormones (irisin, FGF21), cytokines (IL-4/13), natural compounds (berberine, capsaicin), synthetic thermogenic compounds (JAK inhibitors, Notch inhibitors, soluble guanyl cyclase), and endogenous small molecules (serotonin, lactate) [9]. Unfortunately, these compounds have limitations, such as systemic action, the necessity of retreatment to maintain thermogenic phenotype, and some unsolved issues of adipocyte-targeted delivery. In this light, the genetic approach appears to be a considerably more promising avenue for the induction of thermogenic fat. In recent years, numerous research groups have employed diverse technologies, including gene overexpression, microRNA silencing, and genome editing, to generate thermogenic adipocytes [10,11,12]. Additionally, genetic approaches hold promise in addressing the challenge of targeted delivery. The utilization of adipose-specific promoters and viral vectors that exhibit a high degree of affinity for adipocytes may facilitate the generation of modified thermogenic adipocytes for in vivo applications [13,14].

The majority of proposed thermogenic inducers act through the canonical UCP1-dependent thermogenic pathway. However, recent years have yielded substantial progress in the identification of alternative pathways, which have been designated as non-canonical thermogenesis. Classic examples of non-canonical thermogenesis include SERCA2b-dependent thermogenesis and metabolic futile cycling [15]. In a seminal study in 2021, the research group led by Kazak and Spiegelman unveiled a novel futile cycle: the creatine cycle [16,17]. This cycle is characterized by a reciprocal conversion between creatine and phosphocreatine mediated by creatine kinase B (CKB) and tissue nonspecific alkaline phosphatase (TNAP). Consequently, the key players in this recently identified creatine cycle have emerged as promising targets for gene therapy interventions.

While previous studies confirmed the thermogenic role of the creatine cycle and revealed regulatory mechanisms of its activation predominantly in brown adipose tissue [18,19,20,21], we asked whether CKB overexpression in white adipocytes might facilitate thermogenesis and metabolic activity. In the study, we delivered protein-coding CKB sequence into mature adipocytes and subsequently evaluated their bioenergetic properties. The characterization of CKB-overexpressing adipocytes is an important task for the creation of thermogenic adipocytes for gene and cell therapy.

## 2. Methods

### 2.1. Cloning of CKB Protein-Coding Sequence into Lentiviral Transfer Vector

Amplification of the nucleotide sequence encoding CKB was performed by PCR using cDNA derived from mRNA isolated from the mouse brain. Total RNA extraction from C57BL/6J mouse brain was performed using ExtractRNA reagent (Eurogen, Moscow, Russia), followed by purification using CleanRNA Standard kit (Eurogen, Moscow, Russia) according to the manufacturer’s protocols. MMLV RT reverse transcription kit (Eurogen, Moscow, Russia) was used to obtain cDNA. Target sequence amplification was performed by PCR using primers matched to the coding sequence of mouse CKB mRNA NM_021273.4 (Table 1) and High Fidelity Platinum Taq DNA polymerase (Waltham, MA, Invitrogen, USA). PCR was performed in the presence of primers mCKB_clon fwd and mCKB_clon_rev (annealing temperature 60 °C). After amplification, the PCR product was separated by electrophoresis in a 1% agarose gel, followed by gel purification of the target fragment (~1200 bp) using the QIAquick Gel Extraction Kit (Dusseldorf, Qiagen, Germany).

The obtained sequence encoding CKB was cloned into a LeGO-iG2 vector without IRES_GFP (Watertown, MA, Addgene, USA) using restriction sites BamHI and NotI. The cloned sequence was validated by Sanger sequencing (V.A. Engelhardt Institute of Molecular Biology, Russian Academy of Sciences). For further production of lentiviral constructs, the plasmid was amplified and isolated using the QIAGEN Plasmid Maxi Kit (Dusseldorf, Qiagen, Germany).

### 2.2. Lentiviruses Creation for CKB Overexpression

To produce lentiviral particles, calcium-phosphate transfection of HEK293T cells was performed with the assembly plasmids pRSV-Rev (Plasmid #12253, Addgene), pMDLg/pRRE (Plasmid #12251, Addgene), pMD2G (Plasmid #12259, Addgene), and a transfer plasmid encoding CKB (Section 2.1) or green fluorescent protein (for production of control lentivirus; Plasmid #27341, Addgene). Transfection was performed at 70–80% confluency of HEK293T cultured in DMEM HG + 10% FBS medium. A 0.3 M CaCl_2_-based solution containing 7 µg/mL pMDLg/pRRE, 5 µg/mL pRSV-Rev, 3 µg/mL pMD2.G, and 10 µg/mL transfer plasmid was thoroughly mixed with an equal volume of 2 × HBS buffer (40 mM HEPES, 300 mM NaCl, Na_2_HPO_4_ pH 7.1). A volume of 2 mL of solution was added per 100-mm Petri dish. After 18 h of incubation with a transfection mixture, the medium was changed. In 24 h, the conditioned media were collected, centrifuged at 500× *g* for 10 min, the supernatant was collected, and the virus effluent was frozen at −70 °C.

### 2.3. Adipogenic Differentiation

Preadipocyte cell line 3T3-L1 (Manassas, VA, ATCC, USA) was used for the derivation of mature adipocytes. 3T3-L1 was cultivated at 37 °C and 5% CO_2_ in cell culture medium DMEM HG (glucose concentration 4.5 g/L) with supplementation of PenStrep antibiotics mixture (Servicebio, Wuhan, China) and 10% newborn calf serum (LTB, Vilnus, Lithuania). Cell passaging was performed at 80% confluency with Versen solution washing and cell detachment by 0.25% trypsin-EDTA solution. Adipogenic differentiation was performed using the protocol of Zebisch and coauthors [22]; adipogenesis was induced after 100% confluence. Cells were cultured in DMEM HG + 10% FBS (Cytiva, Marlborough, NJ, USA) for 2 days, followed by 2 days in DMEM HG + 10% FBS supplemented by complete adipogenic inducers mix (0.5 mM dexamethasone; 0.25 uM isobutylmethylxanthine; 100 nM insulin; all are from Sigma-Aldrich, St.Louis, MO, USA) and 2 days in DMEM HG + 10% FBS supplemented 100 nM insulin. After that, cells were maintained in DMEM HG + 10% FBS for lipid accumulation. Transduction was performed 4 days after insulin removal, and metabolic tests were carried out 6 days after transduction.

### 2.4. CKB Overexpression in Mature Adipocytes and Estimation of Transgene Expression

For the CKB overexpression, mature adipocytes 3T3-L1 were incubated with lentiviral stocks in a dilution of 1:2 in DMEM HG + 10% FBS supplemented by cationic polymer polybrene in a concentration of 8 mg/mL. The next day, the medium was replaced with a fresh cell culture medium, and the cells were incubated for 6 days with medium replacement every 2 days. The production level of transgene (CKB) was evaluated using real-time PCR and western blotting. RNA was isolated using the CleanRNA Standard kit (Eurogen, Moscow, Russia) and evaluated on the microvolume spectrophotometer NanoDrop2000 (Thermo Scientific, Waltham, MA, USA). cDNA synthesis was performed using MMLV RT kit (Eurogen, Moscow, Russia), RT PCR was performed using SYBR Green-based kit (Syntol, Moscow, Russia) in the system StepOnePlus (Applied Instruments, Indianapolis, IN, USA). Primer sequences were synthesized in Eurogen, Moscow, Russia: CKB forward 5’-AGCACAAGACCGACCTCAAC-3′; reverse 5′-CATCTAGGCTGGACAGAGCTTC-3′; β-actin forward 5′-AGACCTTCAACACCCCAGCCAT-3′; β-actin reverse 5′-GGATGGCGTGAGGGAGAGCATA. mRNA level was determined by 2^(deltaCt) method [23]. For the estimation of CKB protein, cells were lysed in RIPA buffer (50 мM Tris-HCl, pH 8.0, 150 мM NaCl, 1% Triton X-100, 0.5% sodium deoxycholate, and 0.1% sodium laurylsulfate) supplemented with the cOmplete protease inhibitors mix (Roche, Basel, Switzerland). Proteins were separated by the Laemmli SDS-PAGE method [24] with further protein transfer on the PVDF membrane. Membrane was blocked in 5% fat-free milk solution with subsequent staining with primary CKB antibody (A12631, Abclonal, Wuhan, China) and secondary HRP-conjugated antibody (AS014, Abclonal, Wuhan, China). Visualization was carried out using the chemiluminescent kit Clarity ECL (Bio-Rad, Hercules, CA, USA) and the gel-chemidocumenting system Fusion-FX (Vilber Lourmat, Paris, France). Protein band densitometry was performed using GelAnalyzer 2013 software.

### 2.5. Lipid Droplet Analysis

Analysis of lipid droplets (LDs) dynamics was performed using lipophilic dye BODIPY493/503 (Invitrogen, USA). Live cells were stained with 0.25 μg/mL BODIPY493/503 for 20 min at 37 °C and 5% CO_2_. Next, cells were washed thrice with warm PBS, and the medium was replaced with phenol red-free DMEM HG + 10% FBS. Intravital microscopy was performed on a confocal microscope Leica Stellaris 5 (Leica Microsystems, Wetzlar, Germany) supplemented with a temperature- and CO_2_-controlled chamber (Okolab, Pozzuolli, Italy). LD size and number were analyzed by a machine learning algorithm in NIS-Elements software (version 6.10.01, Nikon, Tokyo, Japan) [25]. Each sample was represented by 15 random fields of view.

### 2.6. Thermogenesis Assessment

Mature adipocytes’ thermogenesis was evaluated using thermosensitive dye ERthermAC (Merck, Darmstadt, Germany) according to Kristz et al.’s protocol [26]. Live cells were stained with 250 nM ERthermAC dye for 20 min at 37 °C and 5% CO_2_. Next, cells were washed thrice with warm PBS, and the medium was replaced with phenol red-free DMEM HG + 10% FBS. Before microscopy, cells were acclimated in a temperature- and gas-controlled chamber (Okolab, Italy) at 30 °C for 30 min. Intravital microscopy was performed on the confocal microscope Leica Stellaris 5 (Leica Microsystems, Germany). Each sample was represented by 75 random fields of view obtained using automatic mode in the “Navigator” function.

### 2.7. Glucose Uptake Measurement

For the evaluation of glucose uptake and insulin and catecholamine sensitivity in adipocytes, we applied a non-metabolizable radioactive glucose analogue, [3H]-2-deoxyglucose. Before measurement, adipocytes were stimulated with 100 nM insulin for 20 min or with 10 μM isoproterenol for 2 h. Cells were then washed two times with DMEM without glucose, and further manipulations were also performed in this medium. Cells were incubated in the presence of 10 μM 2-deoxyglucose (Sigma-Aldrich, USA) and 0.5 μCi/mL [3H]-2-deoxyglucose (#ART0103B; American Radiolabeled Chemicals, USA) for 10 min. After that, the cells were washed with ice-cold PBS twice and lysed in RIPA buffer. The samples were added into scintillation liquid Unisolve100 (#95691, KochLight Ltd., Haverhill, UK), and the counts per minute parameter was evaluated on the scintillation counter TRI-CARB 4910TR (PerkinElmer, Springfield, IL, USA). All measurements were performed in three repeats. A volume of 10 μL of cell lysate was used for result normalization by protein concentration analysis using the DC Protein Assay Kit (Bio-Rad, USA).

### 2.8. 14C-Based Metabolic Tracing

The activity of de novo lipogenesis was assessed by incubating adipocytes with labeled U-[14C]-glucose (#ARC0122G; American Radiolabeled Chemicals, Boston, MA, USA) as previously described [27]. Mature adipocytes were incubated in DMEM HG + 10% FBS and 0.5 uCi/mL U-[14C]-glucose for 24 h. Next, cells were washed and lysed in RIPA buffer with consequent lipid extraction in a methanol/chloroform (1:2) mixture. The organic fraction, which contained the total lipid fraction, was dried in a nitrogen stream using Bunsen’s valve. For the 14C incorporation analysis, lipid saponification was performed. Lipid fraction was dissolved in 30% KOH and incubated at 70 °C for 10 min with subsequent addition of an equal volume of 96% ethanol, and the mixture was incubated at 70 °C for 2 h. After saponification, the mixture was neutralised with 6M HCl, and lipids were extracted three times using petroleum ester (fatty acid fraction). The water-soluble fraction contains TAG-derived glycerol and other water-soluble components of complex lipids (TAG-derived glycerol fraction). The fatty acid fraction was dried in a nitrogen stream under Bunsen’s valve and dissolved in a small volume of petroleum ester. All samples were dissolved in scintillation liquid Unisolve100 (#95691, KochLight Ltd., UK), and the counts per minute parameter was evaluated on the scintillation counter TRI-CARB 4910TR (PerkinElmer, USA). All measurements were performed in three repeats. A volume of 10 μL of cell lysate was used for result normalization by protein concentration analysis using the DC Protein Assay Kit (Bio-Rad, USA).

### 2.9. Mitochondrial Potential Analysis

Mitochondrial potential analysis was performed using potential-sensitive dye JC-1 (Thermo Scientific, USA). Principles of JC-1 staining are as follows: aggregation in high-potential mitochondria results in red fluorescence, while accumulation in low-potential mitochondria leads to green fluorescence (λ_ex_ = 485 nm, λ_em1_ = 515–545 nm, λ_em2_ = 580–650 nm). Cells were incubated with 2.5 μM JC-1 in standard culture medium for 20 min, after which cells were washed three times with warm phosphate-salt buffer and placed in DMEM HG + 10% FBS medium without phenol red. Intravital microscopy of mature adipocytes was performed on a confocal microscope, Leica Stellaris 5 (Leica Microsystems, Germany), supplemented with a temperature- and gas-controlled chamber (Okolab, Pittsburgh, PA, USA). Each sample was represented by 15 random fields of view with further analysis in FIJI (version 1.53c) software.

### 2.10. Western Blotting

We have performed Western blotting according to protocol for CKB transgene protein expression analysis (Section 2.4). For the analysis of protein markers, we have used the following primary antibodies: ATGL (#A5126, Abclonal, China), SREBP1 (#A15586, Abclonal, China), Complex V (#ab14748, Abcam, UK), TOMM20 (#ab78547, Abcam, UK), vinculin (#ab18058, Abcam, UK).

### 2.11. Statistical Analysis

Statistical analysis was performed using GraphPad Prism 8.0 software. For pair comparisons, Student’s *t*-test was used. For multiple comparisons, two-way ANOVA with post hoc Tukey test was used. The statistically significant threshold was set at *p* < 0.05. Data are presented as mean ± SEM.

## 3. Results

### 3.1. Mature Adipocytes with CKB Lentivirus Modification Highly Express CKB at Both the mRNA and Protein Level

The first task for every cell genetic modification is the control of transgene production. The most optimal way is to estimate both transcriptional and translational levels.

**Figure 1:**
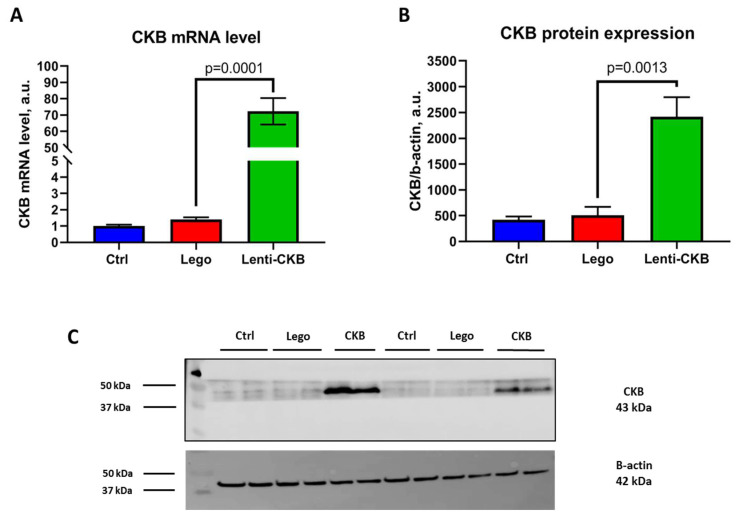
Genetic construct for CKB overexpression enhances the production of CKB mRNA as well as the corresponding protein in 3T3-L1 mature adipocytes. (**A**)—CKB mRNA level, (**B**)—CKB protein expression level, (**C**)—Representative immunoblotting. Data are presented as mean ± standard deviation, n = 4, Student’s *t*-test. Abbreviations: Lego—control lentivirus, Lenti-CKB—lentivirus transferring CKB-coding sequence.

The analysis of mRNA levels revealed a 75-fold augmentation in CKB expression in comparison with the control group and cells modified by control lentivirus (Figure 1A). A similar result was observed for CKB protein levels, which exhibited a fivefold increase compared to the control lentivirus group (Figure 1B,C). These findings substantiate that genetic modification was successful, and CKB-overexpressing adipocytes can be further used for metabolic phenotype description.

### 3.2. CKB Overexpression Does Not Affect Lipid Droplet Number in 3T3-L1 Mature Adipocytes

LD size is one of the most important phenotype parameters of mature adipocytes. In this case, the analysis of LD size during CKB overexpression can indicate changes in the metabolism of adipocytes.

**Figure 2:**
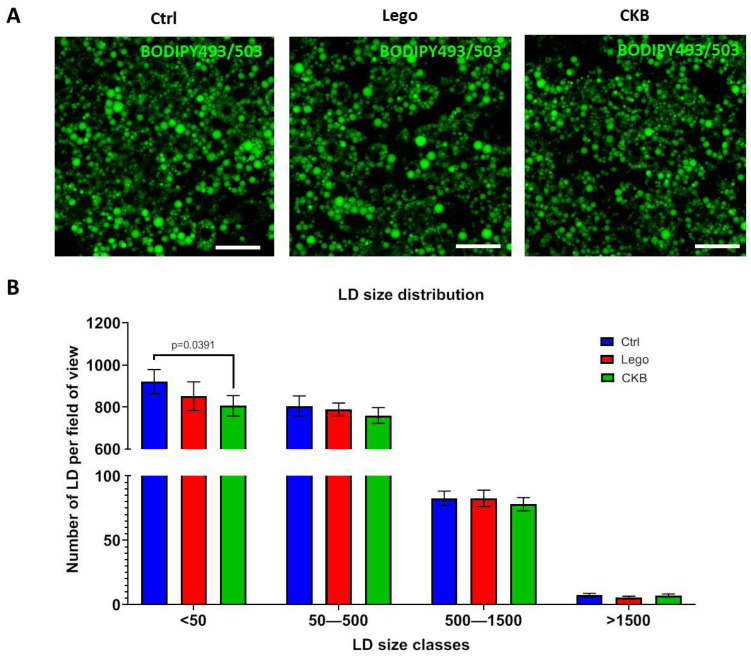
CKB overexpression has no effect on LD size in 3T3-L1 mature adipocytes. (**A**)—Representative images of adipocytes stained with BODIPY493/503 (scale bar—50 μm), (**B**)—Relative LD size distribution. A total of 15 random FOVs were captured for each sample, and the LD distribution was assessed using a deep learning algorithm in NIS-elements software. Data are presented as mean ± standard deviation, ANOVA. Abbreviations: Lego—control lentiviral vector, CKB—vector for CKB overexpression.

The analysis of LD size demonstrated one statistically significant difference: we can see a significant lowering of small LDs in comparison with the control group (Figure 2). However, the LD number in CKB-overexpressed adipocytes did not differ from that of the control lentivirus. In general, we can conclude that CKB overexpression had no effect on LD morphology in mature adipocytes.

The analysis of LD morphology demonstrated that CKB expression has no influence on relative LD size distribution (Figure 2). We can conclude that CKB overexpression does not affect LD dynamics in mature adipocytes.

### 3.3. CKB Overexpression Elevates Glucose Uptake and Reduces TAG Synthesis Under β-Adrenergic Stimulation

In order to characterize the role of CKB in glucose and lipid metabolism in 3T3-L1 mature adipocytes, we further measured glucose consumption, lipogenesis, and sensitivity of cells to the most important metabolic regulators: insulin and β-adrenoreceptor agonist isoproterenol.

**Figure 3:**
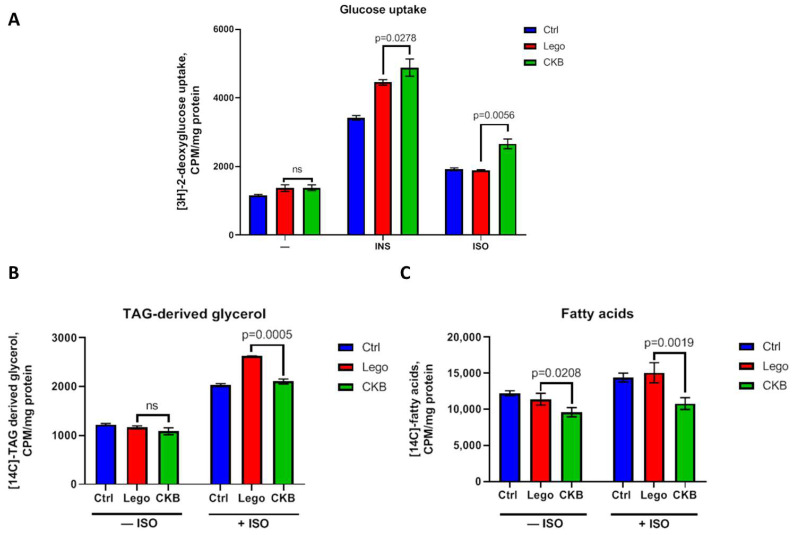
CKB overexpression causes an increase in glucose uptake as well as suppression of TAG synthesis in 3T3-L1 mature adipocytes. (**A**)—Analysis of [3H]-2-deoxyglucose uptake by adipocytes. (**B**)—Incorporation of [14C]-atoms from [14C]-glucose into TAG-derived glycerol. (**C**)—Incorporation of [14C]-atoms from [14C]-glucose into fatty acids. Data are presented as mean ± standard deviation, n = 9, ANOVA. Abbreviations: ISO—isoproterenol, INS—insulin, Lego—control lentiviral vector, CKB—vector for CKB overexpression, ns—non significant difference.

An examination of glucose uptake has revealed that CKB overexpression exhibits no actions in basal conditions. However, CKB overexpression in mature adipocytes significantly enhanced insulin-stimulated and isoproterenol-stimulated (20% increase) glucose uptake (Figure 3A). It can indicate that CKB overexpression can successfully induce bioenergetic processes only under hormonal stimulation, thereby increasing sensitivity to such important metabolic regulators as insulin and catecholamine. CKB expression has been demonstrated to induce alterations in adipocyte lipid metabolism. In basal conditions, CKB expression has been shown to elicit a suppression of fatty acid synthesis. Moreover, under isoproterenol, CKB suppresses both glycerol-3-phosphate and fatty acid synthesis. (Figure 3B,C). In summary, CKB overexpression enhances insulin- and isoproterenol-stimulated glucose uptake in combination with suppression of lipid synthesis. Based on these results, it is imperative to elucidate the metabolic fate of glucose, given its apparent exclusion from lipid synthesis processes. A possible mechanism of glucose utilization besides lipogenesis is complete oxidation in mitochondria.

### 3.4. CKB Overexpression Leads to Mitochondrial Potential Growth in 3T3-L1 Mature Adipocytes

A high level of mitochondrial inner membrane potential is indispensable for efficient ATP synthesis. To understand the state of mitochondria in adipocytes, a potential-sensitive dye, JC-1, was used.

**Figure 4:**
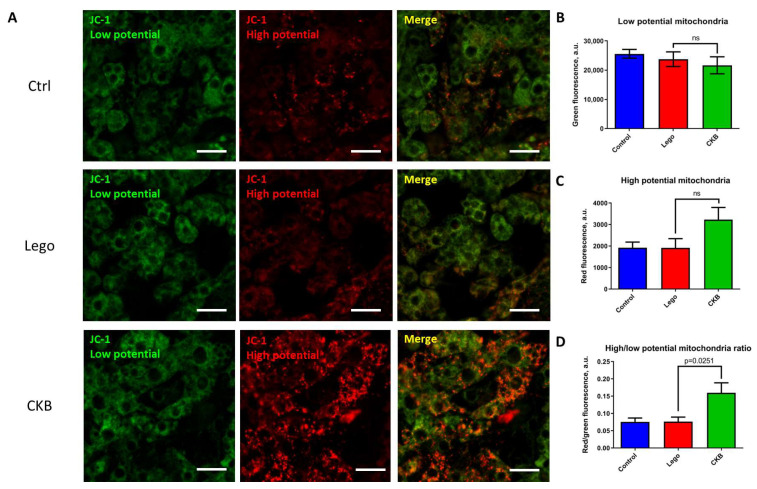
CKB overexpression in 3T3-L1 mature adipocytes increases the number of mitochondria with high membrane potential. (**A**)—Representative images of adipocytes stained with JC-1 (scale bar–50 μm). (**B**)—Quantification of low-potential mitochondria. (**C**)—Quantification of high-potential mitochondria, (**D**)—The ratio of high- to low-potential mitochondria. A total of 15 random FOVs were captured for every sample, and red and green fluorescence were assessed using ImageJ software ((version 1.53c)). Data are presented as mean ± standard deviation, Student’s *t*-test. Abbreviations: Lego—control lentiviral vector, CKB—vector for CKB overexpression, ns – non significant difference.

The analysis has demonstrated an equivalent number of low-potential mitochondria in all groups (Figure 4A,B). The number of mitochondria with high potential tends to increase in cells with high levels of CKB, but this difference is not significant. (Figure 4A,C). However, the ratio of high- to low-potential mitochondria was significantly higher in mature adipocytes with CKB overexpression (Figure 4A,D). Thus, CKB enhances mitochondrial potential in 3T3-L1 mature adipocytes, which may indicate that elevated glucose uptake is related to increased mitochondrial oxidation and ATP synthesis. Further, we suggest that increased ATP demand could be caused by activation of an energy-consuming non-canonical thermogenic mechanism, a part of which is CKB.

### 3.5. CKB Overexpression Suppresses Isoproterenol-Induced Thermogenesis

According to our previous results, we can hypothesize that CKB overexpression may induce thermogenesis. We tested this hypothesis using a temperature-sensitive dye ERthermAC.

**Figure 5:**
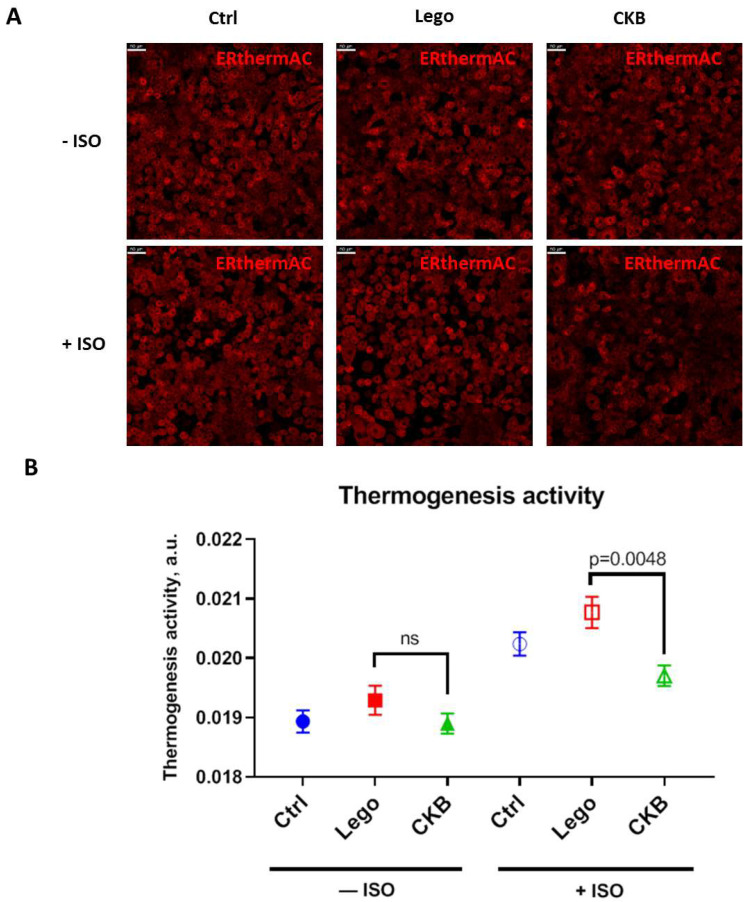
CKB overexpression in 3T3-L1 mature adipocytes suppresses thermogenesis under isoproterenol stimulation. (**A**)—Representative images of adipocytes stained with ERthermAC (scale bar 50 μm). (**B**)—Thermogenesis activity under basal conditionsand isoproterenol stimulation. A total of 75 random FOVs were captured for each sample, and thermogenesis activity was assessed in NIS-elements software using the mean fluorescence intensity mode. Data are presented as mean ± standard deviation, ANOVA. Abbreviations: Lego—control lentiviral vector, CKB—vector for CKB overexpression, ns—non significant difference.

We demonstrated that CKB overexpression in 3T3-L1 mature adipocytes does not alter basal thermogenic activity. Moreover, under isoproterenol, a well-established thermogenic inducer, CKB overexpressing adipocytes exhibited heat production lowering (Figure 5). According to our results, CKB overexpressing 3T3-L1 adipocytes undergo significant metabolic changes without any concomitant elevation of thermogenesis.

### 3.6. Analysis of Protein Markers of Lipid Metabolism and Mitochondrial State in Mature 3T3-L1 Adipocytes with CKB Overexpression

For the confirmation of functional test results and for the evaluation of possible molecular mechanisms involved in CKB overexpression, we have analyzed protein expression of the respective molecular markers.

**Figure 6:**
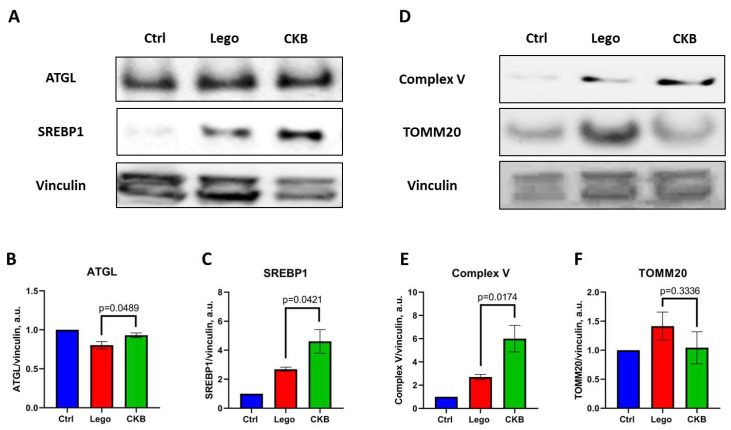
Expression of lipid metabolism and mitochondrial state protein markers in mature 3T3-L1 adipocytes with CKB overexpression. (**A**)—Representative membranes for lipid metabolism markers; (**B**)—Quantitative analysis of ATGL expression; (**C**)—Quantitative analysis of SREBP1 expression; (**D**)—Representative membranes for mitochondrial state markers; (**E**)—Quantitative analysis of Complex V expression; (**F**)—Quantitative analysis of TOMM20 expression. Data are presented as mean ± standard deviation, n = 3, Student’s *t*-test. All western blot membranes were captured from the membrane cut at the molecular markers with the use of stripping; a single loading control was used for one repeat of the experiment for all four target protein markers analysis. Abbreviations: Lego—control lentivirus, CKB—lentivirus transferring CKB—coding sequence.

Using our data about changes in mature adipocytes’ lipid metabolism during CKB overexpression, we have analyzed the expression of some protein markers. ATGL is an adipocyte marker that can regulate basal lipolysis activity. We noticed that CKB overexpression enhances ATGL expression statistically significantly, but the amplitude of this effect was very small (Figure 6A,B). Also, CKB overexpression enhances SREBP1 expression–a transcription factor which can be activated by lipids (Figure 6A,C). It can be a compensatory reaction of lipid synthesis suppression (Figure 3). Mitochondrial state markers demonstrate enhancement in Complex V subunit expression during CKB overexpression, which very accurately correlates with our results about mitochondrial potential (Figure 4 and Figure 6D,E). However, mitochondrial number, which usually correlates with TOMM20 expression, was equal despite CKB overexpression in mature adipocytes (Figure 6D,F). Thus, the results of lipid metabolism and mitochondrial state protein markers analysis confirm our results about mitochondrial potential growth and can reflect a compensatory reaction of SREBP1 expression on lipid synthesis suppression.

## 4. Discussion

Our study demonstrated that CKB overexpression in mature adipocytes leads to glucose uptake and mitochondrial potential growth in combination with fatty acid synthesis suppression. Meanwhile, CKB overexpression in mature adipocytes exerts no effect on LD dynamics and suppresses thermogenesis. In summary, CKB overexpressing adipocytes can be used for nutrient utilization, but not for ectopic thermogenic loci generation. Subsequent discussion will focus on the analysis of potential mechanisms implicated in the observed phenotypic outcomes.

The analysis of LD size distribution revealed no alterations in LD dynamics in response to CKB expression in mature adipocytes (Figure 2). This finding is consistent with the hypothesis that CKB-driven thermogenesis does not depend on LD degradation, given that triglycerides are not the only source of energy for ATP generation. Furthermore, CKB is a crucial enzyme in adipocytes, which is indispensable for active mitochondrial ATP synthesis. Suppression of the CKB gene results in adipocytes becoming glycolytic [19]. Thus, we confirm that CKB overexpression does not enhance lipid hydrolysis.

Glucose uptake analysis showed an increase in insulin- and isoproterenol-stimulated glucose consumption in adipocytes (Figure 3A). Specific glucose uptake growth in insulin-stimulated conditions can be related to possible CKB phosphorylation by Akt kinase, which is activated by insulin [20]. CKB can also be activated by isoproterenol, because isoproterenol has an action on α1-adrenoreceptor, and α1-adrenoreceptor–Gαq signalling potentiates adipocyte thermogenesis through CKB and TNAP [21,28]. Moreover, we have noticed SREBP1 expression growth in mature adipocytes with CKB overexpression (Figure 6), which can enhance glycolysis and drive glucose metabolism. In summary, the growth of insulin- and isoproterenol-stimulated glucose uptake can be related to the action of respective molecules on CKB and can drive CKB-dependent thermogenesis.

Next, we analysed the metabolic fate of glucose by measurement of [14C]-atoms incorporation from labeled glucose to TAG-derived glycerol (glyceroneogenesis, from glycolysis intermediates) and to fatty acids (de novo synthesis). Our results demonstrated that in basal conditions, CKB overexpression leads to suppression of fatty acid synthesis, whereas in isoproterenol-stimulated adipocytes with CKB overexpression, there is a downregulation in both TAG-derived glycerol and fatty acid synthesis (Figure 3B,C). This may indicate that CKB overexpression, especially under isoproterenol-stimulated conditions, leads to an increase in ATP demand in adipocytes and that the taken-up glucose is used for ATP production.

To test this, we have analyzed mitochondrial potential as an essential parameter for ATP synthesis. Our results showed an increase in the ratio of high- to low-potential mitochondria in mature adipocytes with CKB overexpression (Figure 4). This is consistent with recent studies showing that CKB knockdown impairs mitochondrial respiration and decreases membrane potential [16]. Our data about mitochondrial potential can also be confirmed by the detected growth of ETC Complex V subunit expression (Figure 6). It seems that CKB overexpression leads to an increase in ATP demand, which is compensated by an increase in glucose uptake and complete oxidation.

One of the energy-consuming processes in CKB overexpressing adipocytes could be the activation of thermogenic creatine futile cycling. Unexpectedly, our findings demonstrate that CKB overexpression has no influence on basal thermogenesis and ulates isoproterenol-stimulated heat production (Figure 5). A potential explanation for the observed decrease in thermogenesis in CKB-overexpressing adipocytes is the low activity of TNAP, which is the second component of the CKB/TNAP futile cycle. However, the available evidence regarding TNAP activity appears to have minimal impact on this observation. This is due to the fact that TNAP activity in adipocytes has been shown to exhibit a strong correlation with enhanced lipid metabolism, a finding that has not been previously documented [29,30]. The second potential reason for the lack of CKB-driven thermogenesis is the inactive enzyme state. Despite the high level of CKB expression observed in genetically modified mature adipocytes in our study, it does not necessarily reflect the corresponding enzymatic activity of CKB. CKB has at least four phosphorylation sites and one nitrosylation site, which can regulate CKB activity [31,32,33]. The regulation of CKB and TNAP activity in adipocytes is a promising avenue for future research.

In summary, CKB overexpression in mature adipocytes allows us to obtain adipocytes with high stimulated glucose uptake, elevated ATP synthesis, and suppressed lipid metabolism—i.e., augmented ATP synthesis and repressed lipid metabolism. It is important to note that CKB expression does not initiate the thermogenesis process. However, CKB may support other processes with high energy demand in mature adipocytes.

## 5. Conclusions

In this study, we demonstrated that the expression of CKB in mature adipocytes results in cells with elevated glucose uptake, augmented ATP synthesis, and repressed lipogenesis. These genetically modified cells may potentially exhibit a positive metabolic effect in the context of excessive nutrient utilization. However, further investigation is necessary to elucidate the mechanisms by which CKB overexpression affects adipocyte physiology.

## Figures and Tables

**Table 1:** Primer sequences for amplification of the protein-coding sequence of glycerol kinase. Underlined: sequence complementary to NM021273.4; bold: Kozak sequence; grey: BamHI and NotI restriction sites.

Primer Type	Sequence	Annealing Temperature
mCKB_clon fwd	ATATA GGATCC **TGCCGCCGCC **ATGC	61.7 °C
mCKB_clon_rev	ATT GCGGCCGC TCACTTCTGG GCCGGCAT	59.7 °C

## Data Availability

The datasets generated and analyzed during the current study are available from the corresponding author on reasonable request.

## Abbreviations

ATP—adenosine triphosphate; ANOVA—analysis of variance; CKB—creatine kinase B; CoA—coenzyme A; DMEM HG—Dulbecco’s modified Eagle medium; EDTA—ethylenediaminetetraacetic acid; FGF21—fibroblast growth factor type 21; FBS—fetal bovine serum; HRP—*Horseradish* sp. peroxidase; iSGLT2—inhibitors of sodium-glucose transporter type 2; IL-4/13—interleukins type 4/13; JAK—Janus kinase; LD—lipid droplets; PCR—polymerase chain reaction; PVDF—polyvinylidene difluoride; SERCA2b—sarco/endoplasmic reticulum Ca^2+^-ATPase; SDS PAGE—sodium dodecyl sulfate polyacrylamide gel; RIPA buffer—radioimmunoprecipitation assay buffer; TAG—triacylglycerides; TNAP—tissue nonspecific alkaline phosphatase; UCP-1—uncoupling protein 1.
